# Clinical Findings and Genetic Expression Profiling of Three Pigmented Lesions of the Optic Nerve

**DOI:** 10.1155/2015/590659

**Published:** 2015-04-23

**Authors:** Manuel A. de Alba, Victor M. Villegas, Aaron S. Gold, Andrea Wildner, Fiona J. Ehlies, Azeema Latiff, Timothy G. Murray

**Affiliations:** Murray Ocular Oncology and Retina, 6705 Red Road, Suite 412, Miami, FL 33143, USA

## Abstract

*Background*. Optic disk melanocytoma is a primary tumor of the optic disk that represents a clinical diagnostic challenge due to its similarities with melanoma. *Purpose*. The authors present three cases in which genetic expression profiling was used to identify tumor prognosis of optic disk melanocytoma. *Case Series*. In two cases fine-needle aspiration biopsy was performed to obtain tissue through a transvitreal route into the apex of the tumor while the patient underwent pars plana vitrectomy, laser ablation, phacoemulsification with posterior chamber intraocular lens implantation, and intravitreal triamcinolone acetonide. In the other case the tissue was obtained after definite enucleation. *Conclusion*. Genetic expression profiling is a useful diagnostic tool for classification and can provide vital information to the ocular oncologist regarding prognosis.

## 1. Introduction

Optic disk melanocytoma is a rare benign primary pigmented tumor of the optic disk characterized clinically as a dark brown to black lesion with feathery margins [1–6]. Melanocytomas represent a diagnostic challenge because of the clinical similarities with melanoma [5, 7–9]. Some patients have been treated with enucleation due to confusion between both pathologies. Optic disk melanocytomas have a typical clinical appearance and tend to have benign course. Even though in most of the cases these lesions remain stable, progressive growth and malignant transformation have been documented [8, 10]. Close follow-up is essential to document growth. Fundus photos, autofluorescence, B-scan ultrasonography, spectral-domain optic coherence tomography (SD-OCT), and fluorescein angiography are important for diagnosis and management [1, 11, 12]. Low reflectivity during A-scan testing is suspicious for malignant transformation. The most common treatment for malignant lesion is enucleation.

We report three patients with optic nerve melanocytomas that underwent fine-needle aspiration biopsy and genetic expression profile assessment. Patients were evaluated between 2012 and 2014 (Table 1). All patients underwent a comprehensive ophthalmologic evaluation and follow-up with fundus photos, A/B-scan ultrasonography, and optical coherence tomography.

## 2. Case Series

### 2.1. Case  1

**Figure 1:**
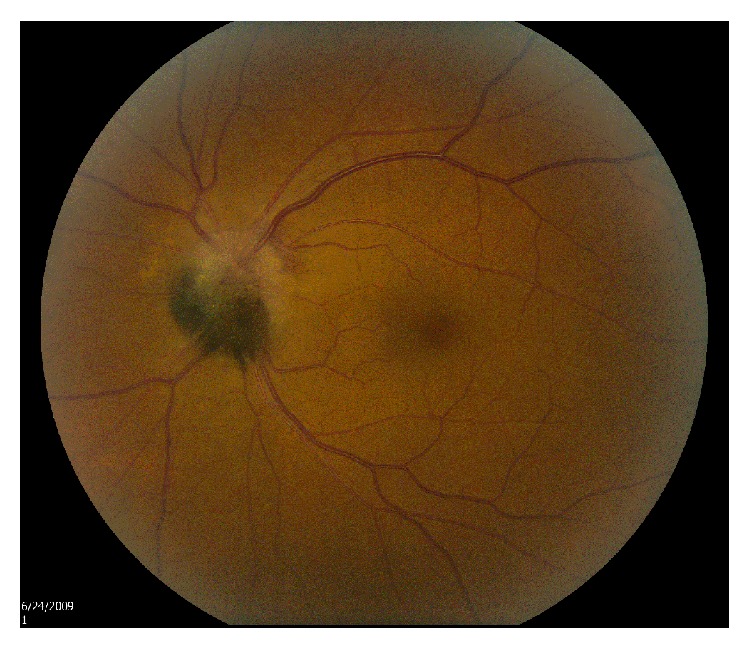
Case  1: fundus photograph of optic disk melanocytoma.

**Figure 2:**
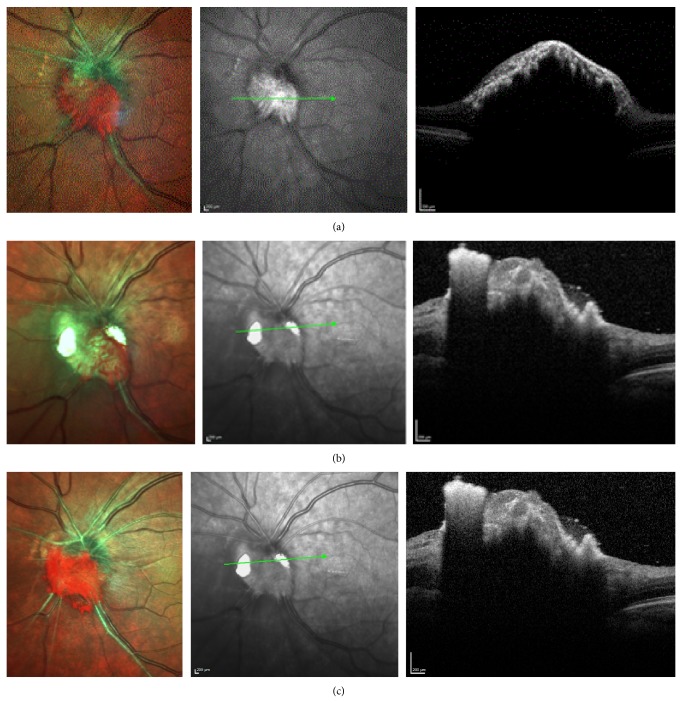
Case  1: fundus multicolor montage photograph and SD-OCT of optic disk melanocytoma fine-needle aspiration biopsy site are shown above. (a) Preoperative imaging of tumor: multicolor, infrared, and SD-OCT. (b) Postoperative day 1. Note the hyperreflective triamcinolone acetonide at the vitreoretinal interface with posterior shadowing. (c) Three months after surgery. SD-OCT shows reduction in tumor volume and no vitreoretinal interface defect.

A 71-year-old Caucasian woman presented to our clinic with obscurations in the left eye (OS). On examination, her best-corrected visual acuity was 20/25 in both eyes (OU). Intraocular pressure was 11 mmHg in the right eye (OD) and 15 mmHg OS. Anterior segment was unremarkable except for moderate nuclear sclerosis OU. Pupils were round and equally reactive to light without an afferent pupillary defect. Dilated fundus examination revealed a highly pigmented, brown, papillary lesion with overlying small drusen OS (Figure 1). The mass measured 3 mm × 2 mm. Ultrasonographic examination revealed an apical height of 1.2 mm with medium to high internal reflectivity. There were no marked signs of malignant transformation or vascular activity. As cataract progressed, pars plana vitrectomy (PPV), phacoemulsification with posterior chamber intraocular lens (IOL) implantation, membrane peel (MP), intravitreal triamcinolone acetonide (IVT), and laser ablation (LA) of the tumor with fine-needle aspiration biopsy (FNB) of the optic disk melanocytoma were performed. Gene expression profile assessment was undertaken at Castle Biosciences Inc. Postoperative follow-up included multicolor fundus photography and SD-OCT (Heidelberg) of the tumor and biopsy incision site (Figure 2). Genetic expression profiling revealed a class 1A genetic signature.

### 2.2. Case  2

**Figure 3:**
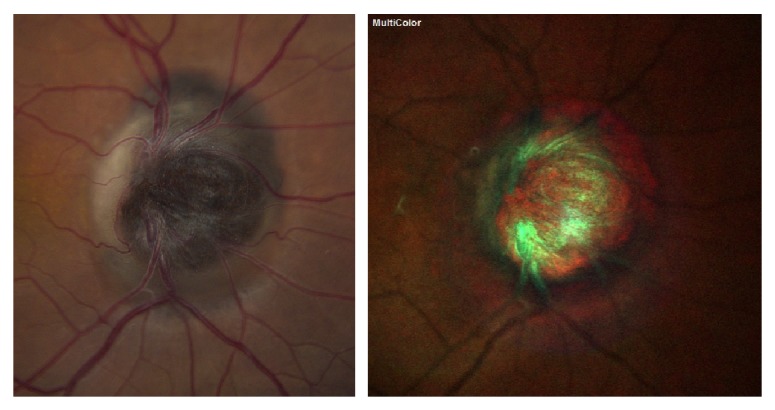
Optic disk photograph and multicolor showing a deeply pigmented lesion in the optic nerve head with adjacent choroid and retinal involvement.

**Figure 4:**
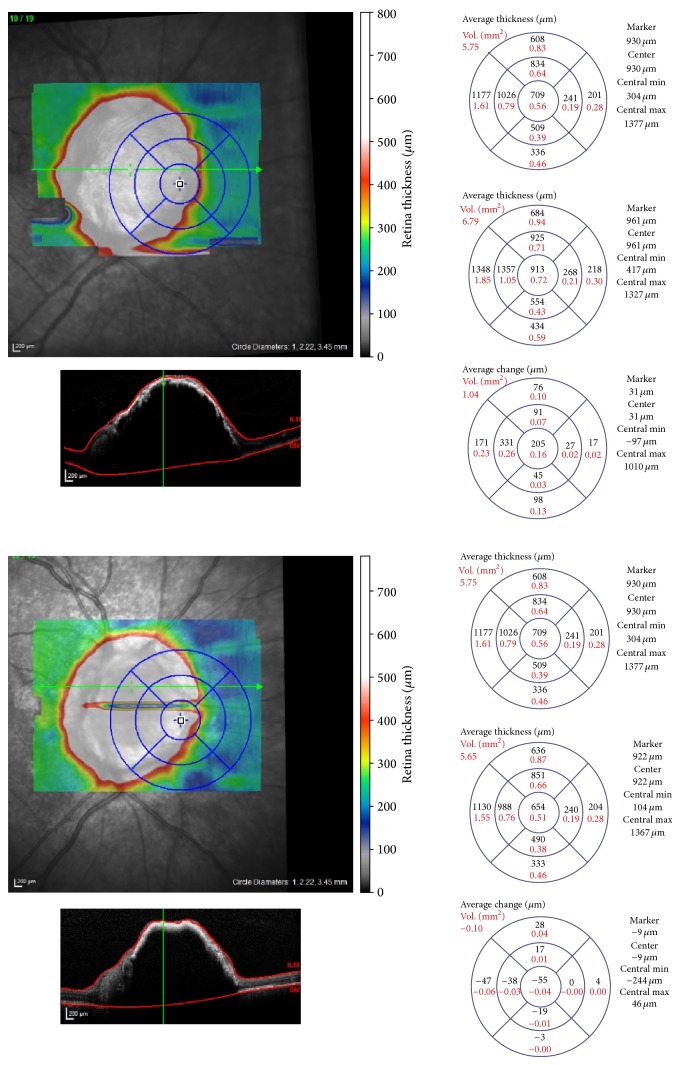
Thickness maps before and after surgery.

A 45-year-old Hispanic woman was referred to the clinic for evaluation of an atypical optic nerve head melanocytoma OD. On examination, her best-corrected visual acuity was 20/50 OD and 20/20 OS. Intraocular pressure was 14 mmHg in both eyes. Visual acuity progressively decreased over the next year to the level of 20/200 secondary to cataract and optic nerve head compression. A relative afferent papillary defect developed OD. Visual field constriction OD was present. Dilated fundus examination OD revealed a deeply pigmented lesion in the optic nerve head with adjacent choroid and retinal involvement (Figure 3). The tumor measured 5 mm × 5 mm in diameter. Echographic examination revealed tumor thickness of 2.1 mm. A-scan showed medium reflectivity. As cataract progressed and visual acuity decreased to 20/200, pars plana vitrectomy, phacoemulsification with posterior chamber intraocular lens implantation, membrane peel, intravitreal triamcinolone acetonide, and laser ablation of the tumor with fine-needle aspiration biopsy of the optic disk melanocytoma were performed. Genetic expression profiling was a class 1A. Best-corrected visual acuity was 20/100 at 1 month after surgery. SD-OCT thickness maps (Figure 4) show mild reduction of tumor volume after treatment.

### 2.3. Case  3

**Figure 5:**
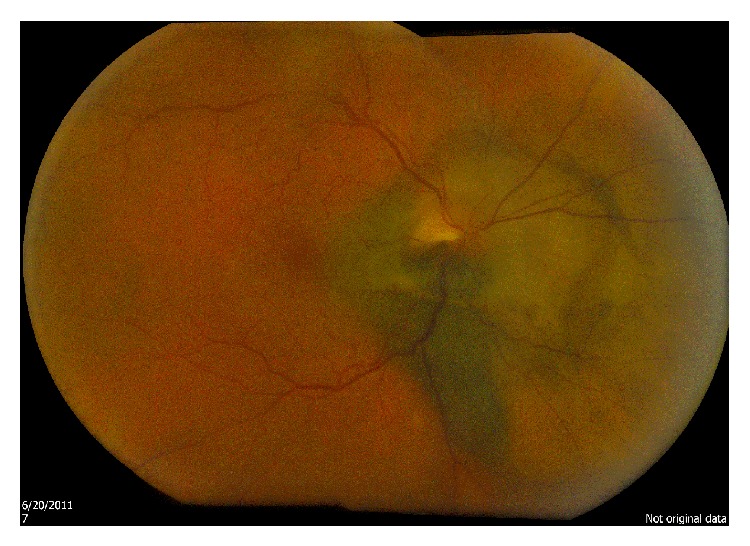
Fundus photograph at initial presentation upon examination.

**Figure 6:**
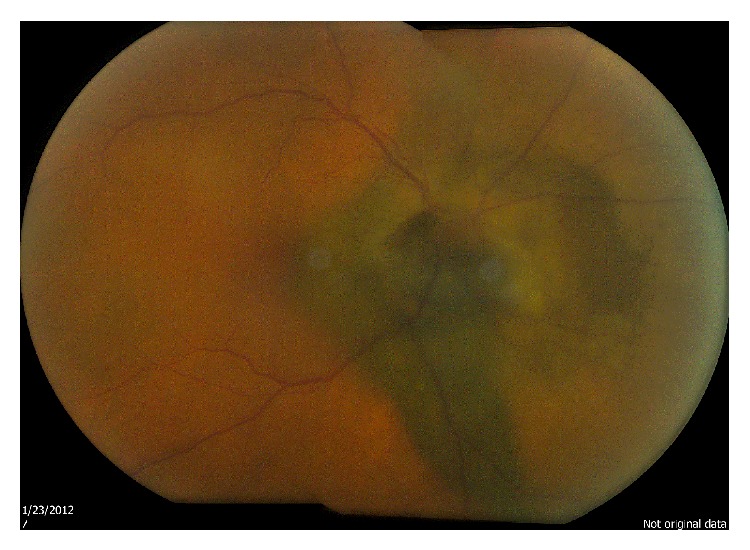
Fundus photograph after 6 months.

A 74-year-old Caucasian male presented to our clinic with an atypical optic disk melanocytoma with associated subretinal fluid and neovascular activity OD. At examination, his best-corrected visual acuity was 20/200 OD and 20/20 OS. Fundus dilation revealed a choroidal papillary and peripapillary pigmented mass with associated subretinal fluid (Figure 5). The lesion base measured 9.5 mm × 7.5 mm. Apical height was 1.7 mm by ultrasonographic testing. Close follow-up revealed enlargement over a 6-month period. Enucleation was performed due to growth and malignant transformation (Figure 6). Histopathology report showed melanocytic malignant melanoma and a genetic expression profile of class 1A. No evidence of metastasis has been present after 2 years of follow-up.

## 3. Discussion

Optic nerve melanocytoma is a deeply pigmented elevated lesion within the optic nerve head and often involving nerve fiber layer of the adjacent retina [4, 10, 12]. It is most commonly located at the inferotemporal quadrant of the optic disk [10]. These lesions are commonly unilateral, although bilateral cases have been reported in children [13, 14]. Melanocytomas are often an incidental finding in routine ophthalmological evaluation. The mean age of diagnosis is in the sixth decade of life [2, 14], with a predilection for females [3, 14] and equal distribution within races [14, 15]. The pathogenesis of these lesions is unknown and is usually a local pathology with no association with systemic diseases [14].

Visual prognosis is excellent in most of the cases [2, 3, 14], but decreased visual acuity may occur in rare cases. Visual loss can result from secondary optic neuropathy [6, 14], optic disk edema [1, 11], spontaneous tumor necrosis [3, 14, 16], juxtapapillary or foveal choroidal neovascularization [1, 11, 12, 14], subretinal fluid from tumor exudation [6], cystoid retinal edema [1], retinal traction [1], epiretinal membrane [1], central retinal vein occlusion [5, 16], or malignant transformation [12, 14].

Although melanocytomas do not typically grow [2, 4, 14], slow progressive enlargement has been documented in 10 to 15% of the cases [4, 6, 17]. Growth is not necessarily a sign of malignant transformation [2, 4].

When extensive involvement of the optic nerve is present and rapid progressive visual loss occurs, malignant transformation should be suspected. However, optic nerve ischemia secondary to spontaneous tumor necrosis cannot be ruled out [12, 17]. It is important to differentiate optic nerve melanocytoma from other pigmented optic nerve head lesions, such as juxtapapillary choroidal melanoma, choroidal nevus, hyperplasia of the RPE, combined hamartoma of the retina and RPE and adenoma of the RPE, primary metastatic melanoma to optic disk, and peripapillary vitreous hemorrhage [6, 14].

Fundus photography to monitor growth and morphological changes of the lesion is essential in every examination. SD-OCT provides high-resolution imaging that helps to characterize the morphology of the tumor, detects pathologic vascular activity, and assists in the evaluation for growth [6, 18].

Transscleral and transretinal fine-needle aspiration have been used for cytopathology of intraocular tumors and for genetic material analysis [19–22]. In recent years, transretinal fine-needle aspiration biopsy during pars plana vitrectomy has been used safely and effectively to obtain tissue sample for risk classification of small melanocytic choroidal tumors with genetic expression profile analysis [19, 23]. In cases  1 and  2, a 25-gauge needle was passed into the apex of the tumor after removal of the hyaloid. Intravitreal triamcinolone acetonide was administered at the end of the procedure to minimize inflammation [19]. Murray et al. showed the use of spectral-domain optic coherence tomography during postoperative examination to highlight the fine-needle aspiration biopsy incision site of posterior segment incision wound architecture for intraocular tumors [19]. In case  1 spectral-domain optic coherence tomography shows the biopsy incision site of the optic nerve tumor with a hyporeflective wound gape with adjacent tissue displacement. To our knowledge, this is the first report documenting fine-needle aspiration incision wound architecture of optic nerve lesions with spectral-domain optic coherence tomography. Gene expression profiling has been used to study DNA of melanoma and classify the tumor genetics into class 1a, 1b, or 2 [19, 24]. Tumor class stratification helps predict cancer prognosis assessing tumor aggressiveness and possibility of metastasis [23]. It is the first time melanocytoma has been assessed with gene expression profiling as well. All cases were class 1a, including case  3 which showed clinical transformation. Further studies are needed to understand the clinical importance of the results.

## 4. Conclusions

Optic nerve melanocytoma has typical characteristics, but it may be confused both clinically and histopathologically with malignant melanoma. Adequate ophthalmological examination, close follow-up visits, and the use of modern ancillary testing must be performed periodically on these patients in order to avoid misdiagnosis and avoid erroneous enucleation. SD-OCT provides excellent visualization of the incision site in the postoperative period. Genetic expression profiling is a useful diagnostic tool for classification and can provide vital information for the ocular oncologist regarding prognosis.

## Figures and Tables

**Table 1:** 

	Patient 1	Patient 2	Patient 3
Age	71	45	74
Sex	Female	Female	Male
Race	Caucasian	Hispanic	Caucasian
Eye	Left eye	Right eye	Right eye
Visual acuity before treatment	20/25	20/200	20/400
Tumor size	3 × 2 × 1.2 mm	5 × 5 × 2.1 mm	10 × 8 × 2.3 mm
Treatment	PPV/MP/phaco./IOL/FNB/LA/IVT	PPV/MP/phaco./IOL/FNB/LA/IVT	Enucleation
Gene expression profiling	1A	1A	1A
Final visual acuity	20/20	20/100	—

## References

[1] Eldaly H., Eldaly Z. (2014). Melanocytoma of the optic nerve head, thirty-month follow-up. *Seminars in Ophthalmology*.

[2] Shields J. A., Demirci H., Mashayekhi A., Shields C. L. (2004). Melanocytoma of optic disc in 115 cases: the 2004 Samuel Johnson Memorial Lecture, part 1. *Ophthalmology*.

[3] Zimmerman L. E., Garron L. K. (1962). Melanocytoma of the optic disk. *International Ophthalmology Clinics*.

[4] Joffe L., Shields J. A., Osher R. H., Gass J. D. M. (1979). Clinical and follow-up studies of melanocytomas of the optic disc. *Ophthalmology*.

[5] Zografos L., Othenin-Girard C. B., Desjardins L., Schalenbourg A., Chamot L., Uffer S. (2004). Melanocytomas of the optic disk. *American Journal of Ophthalmology*.

[6] Punjabi O. S., Lin C. F., Chung H.-S., Gill M. K. (2011). Melanocytoma of the optic disc associated with visual field defects: clinical features and imaging characteristics. *Ophthalmic Surgery, Lasers & Imaging*.

[7] Le Z. (1960). Pigmented tumors of the optic nerve head—the 22nd Annueal de Schweinitz Lecture. *American Journal of Ophthalmology*.

[8] De Potter P., Shields C. L., Eagle R. C., Shields J. A., Lipkowitz J. L. (1996). Malignant melanoma of the optic nerve. *Archives of Ophthalmology*.

[9] Erzurum S., Lee J., Territo C., O'Grady R. (1992). Primary malignant melanoma simulating a melanocytoma optic nerve. *Archives of Ophthalmology*.

[10] Meyer D., Ge J., Blinder K. J., Sinard J., Xu S. (1999). Malignant transformation of an optic disk melanocytoma. *American Journal of Ophthalmology*.

[11] Al-Rashaed S., Abboud E. B., Nowilaty S. R. (2010). Characteristics of optic disc melanocytomas presenting with visual dysfunction. *Middle East African Journal of Ophthalmology*.

[12] Mohmad Z., Aik Kah T., Chui Yong K., Wan Abdul Halim W. H., Kong Yong T. (2011). Melanocytoma of the optic nerve head—a diagnostic dilemma. *Clinics and Practice*.

[13] Walsh T. J., Packer S. (1971). Bilateral melanocytoma of the optic nerve associated with intracranial meningioma. *Annals of Ophthalmology*.

[14] Shields J. A., Demirci H., Mashayekhi A., Eagle R. C., Shields C. L. (2006). Melanocytoma of the optic disk: a review. *Survey of Ophthalmology*.

[15] Phillpotts B. A., Sanders R. J., Shields J. A., Griffiths J. D., Augsburger J. A., Shields C. L. (1995). Uveal melanomas in black patients: a case series and comparative review. *Journal of the National Medical Association*.

[16] Shields J. A., Shields C. L., Eagle R. C., Singh A. D., Berrocal M. H., Berrocal J. A. (2001). Central retinal vascular obstruction secondary to melanocytoma of the optic disc. *Archives of Ophthalmology*.

[17] Gupta V., Gupta A., Dogra M. R., Pandav S. S. (1995). Progressive growth in melanocytoma of the optic nerve head. *Indian Journal of Ophthalmology*.

[18] Shields C. L., Perez B., Benavides R., Materin M. A., Shields J. A. (2008). Optical coherence tomography of optic disk melanocytoma in 15 cases. *Retina*.

[19] Murray T. G., Gold A. S., Markoe A. M. (2014). Spectral-domain optical coherence tomography evaluation of choroidal melanoma and nevus fine-needle aspiration biopsy incision sites. *Ophthalmic Surgery, Lasers and Imaging Retina*.

[20] Young T. A., Burgess B. L., Rao N. P., Glasgow B. J., Straatsma B. R. (2008). Transscleral fine-needle aspiration biopsy of macular choroidal melanoma. *American Journal of Ophthalmology*.

[21] McCannel T. A., Chang M. Y., Burgess B. L. (2012). Multi-year follow-up of fine-needle aspiration biopsy in choroidal melanoma. *Ophthalmology*.

[22] Shields C. L., Ganguly A., Materin M. A. (2007). Chromosome 3 analysis of uveal melanoma using fine-needle aspiration biopsy at the time of plaque radiotherapy in 140 consecutive cases. *Transactions of the American Ophthalmological Society*.

[23] Gold A. S., Murray T. G., Markoe A. M. (2014). Uveal melanoma gene expression status post radiotherapy. *Optometry and Vision Science*.

[24] Onken M. D., Worley L. A., Ehlers J. P., Harbour J. W. (2004). Gene expression profiling in uveal melanoma reveals two molecular classes and predicts metastatic death. *Cancer Research*.

